# Body mass index mediates the relationship between depression and triglyceride levels: Evidence from a large national cohort

**DOI:** 10.1016/j.jad.2025.119889

**Published:** 2025-07-11

**Authors:** Bhaavyaa B. Shah, Michael L. Thomas, Michael J. McCarthy, Alejandro D. Meruelo

**Affiliations:** a University of California, San Diego, 9500 Gilman Dr, La Jolla, CA 92093, USA; b Colorado State University, 1876 Campus Delivery, Fort Collins, CO 80523-1876, USA; c University of California, San Diego, VA San Diego Healthcare System, 3350 La Jolla Village Dr, San Diego, CA 92161, USA; d University of California, San Diego, 9500 Gilman Dr, La Jolla, CA 92093, USA

**Keywords:** Depression, Body mass index, Triglycerides, HDL cholesterol, LDL cholesterol, Mediation analysis

## Abstract

**Background:**

Major Depressive Disorder (MDD) is linked to adverse metabolic outcomes, including elevated triglycerides and body mass index (BMI). However, mechanisms underlying this relationship—and their variation by age or sex—remain unclear.

**Methods:**

Using data from over 200,000 participants in the All of Us Research Program, we examined associations between MDD (defined by SNOMED-coded diagnoses), BMI, and lipid biomarkers (triglycerides, HDL, LDL). Causal mediation analyses tested whether BMI mediated the MDD–triglyceride relationship, adjusting for age, sex, and alcohol intake. Sex differences and age-stratified effects (ages 25, 45, 65) were also explored.

**Results:**

Individuals with MDD had higher BMI and triglyceride levels than those without (BMI SMD = 0.234; triglycerides SMD = 0.195; both *p* < 0.001). BMI significantly mediated the MDD–triglyceride association (ACME = 5.46, 95 % CI [2.95, 8.17], *p* < 0.001), accounting for 34.1 % of the total effect. The direct effect was not statistically significant (ADE = 10.55, *p* = 0.084). Mediation effects were consistent across sexes. Stronger mediation was observed at older ages (e.g., ACME = 6.07 at age 65, *p* < 0.001) but not at younger ages (e.g., ACME = 5.09 at age 25, *p* = 0.076). Multiple imputation analyses confirmed these findings (ACME = 3.71, 95 % CI [3.09, 4.33], p < 0.001; proportion mediated = 24.9 %). HDL and LDL levels differed modestly by MDD status.

**Conclusions:**

BMI partially mediates the relationship between MDD and triglycerides, particularly in older adults. These results support integrating metabolic risk management into mental health care.

## 1. Introduction

Major Depressive Disorder (MDD) is one of the most prevalent and disabling psychiatric conditions worldwide, with broad implications for both mental and physical health (Cui et al., 2024). In addition to its emotional and cognitive toll, MDD is strongly associated with increased risk for cardiometabolic disease, including obesity, insulin resistance, and dyslipidemia (Egede, 2004; Pan et al., 2012). These comorbidities are not only clinically burdensome but may also share overlapping biological mechanisms with MDD, such as hypothalamic-pituitary-adrenal (HPA) axis dysregulation (Lopez-Duran et al., 2009), circadian misalignment (Barandas et al., 2015), chronic low-grade inflammation (Dowlati et al., 2010), and adverse health behaviors—including reduced physical activity, poor dietary intake, cigarette smoking, and excessive alcohol use (Kunugi, 2023; Schuch et al., 2018; Selvaraj et al., n.d.).

Among metabolic abnormalities, elevated triglycerides have emerged as a particularly robust biomarker of cardiovascular risk in individuals with MDD. Elevated triglyceride levels have been observed consistently across diverse populations with depression, even after controlling for lifestyle factors (Han, 2022; Lemche et al., 2017; Xu et al., 2024). High triglyceride levels reflect increased hepatic very-low-density lipoprotein (VLDL) production and impaired lipoprotein lipase activity, both of which may be exacerbated by behavioral and neuroendocrine changes in depression—such as emotional eating, HPA axis activation, and pro-inflammatory cytokine production (Milaneschi et al., 2019). However, findings for other lipid fractions, such as high-density lipoprotein (HDL) and low-density lipoprotein (LDL) cholesterol, have been more mixed (Bharti et al., 2021; van Reedt Dortland et al., 2013), raising questions about lipid-specific pathways and potential explanatory factors.

Body mass index (BMI), a widely used marker of adiposity, may serve as a key explanatory variable linking MDD to dyslipidemia—particularly to triglyceride elevation (Fulton et al., 2022; Huang et al., 2023; Kraus et al., 2023). Depression is associated with higher BMI through several behavioral and biological pathways, including increased emotional eating (Konttinen et al., 2019; Simmons et al., 2016), physical inactivity (Koster et al., 2010; Staiano et al., 2016), and neuroendocrine alterations that affect appetite and energy regulation (Milaneschi et al., 2019). In turn, higher BMI—especially increased visceral adiposity—is known to upregulate lipolysis and hepatic triglyceride synthesis, leading to elevated circulating triglyceride levels (Grundy, 2004; Klop et al., 2013). Thus, BMI may act as a biological and behavioral mediator between MDD and triglyceride levels.

While this mediation hypothesis is biologically plausible, it has rarely been tested formally using large, population-based data. Furthermore, the potential for BMI to mediate MDD associations with other lipids, such as HDL and LDL cholesterol, remains underexplored. Notably, HDL cholesterol is often reduced in individuals with obesity and metabolic syndrome, whereas LDL levels may be less sensitive to changes in adiposity and more influenced by genetic or dietary factors (Lamarche et al., 1998). These differences highlight the importance of examining lipid subtypes separately when considering depression-related dyslipidemia.

Sex differences further complicate these relationships. Men and women differ in body fat distribution, hormonal regulation, and lipid metabolism, which may contribute to sex-specific cardiometabolic responses to MDD (Goldstein et al., 2019; Lumish et al., 2020). For instance, MDD may promote visceral fat accumulation more prominently in men and subcutaneous fat in women, potentially influencing lipid profiles in divergent ways (Karastergiou et al., 2012). Despite these biological differences, few studies have examined whether BMI-mediated effects of MDD on lipid profiles vary by sex.

To address these gaps, we analyzed data from the All of Us Research Program (“The ‘All of Us’ Research Program”, 2019), a large, diverse national cohort, to test whether BMI mediates the association between MDD and three key lipid biomarkers: triglycerides, HDL cholesterol, and LDL cholesterol. We also investigated whether these mediation pathways differed by sex. We hypothesized that (1) MDD would be associated with higher BMI and adverse lipid profiles; (2) BMI would partially mediate the association between MDD and each lipid outcome, particularly for triglycerides; and (3) mediation effects would be stronger in men, given sex differences in fat distribution and metabolic risk.

Although prior research has linked depression to BMI and triglycerides, few studies have formally tested these mediating pathways across multiple lipid fractions in large, representative cohorts. We additionally conducted exploratory age-stratified mediation analyses to examine whether indirect effects of MDD on triglycerides via BMI vary across the adult lifespan. This study aims to clarify mechanistic links between psychiatric and metabolic health using a rigorous mediation framework applied to one of the largest available real-world datasets.

## 2. Methods

### 2.1. Study population

We used data from the All of Us Research Program (Registered Tier Dataset v8) (“The ‘All of Us’ Research Program”, 2019), focusing on adults aged 18 years and older. Participants were included if they had at least one recorded measurement for triglycerides, cholesterol markers, body mass index (BMI), and a structured diagnosis of MDD. When multiple values were available for the same biomarker or BMI, we retained only the most recent non-missing measurement per participant to reduce measurement redundancy and ensure temporal consistency across variables. Implausible values (e.g., BMI <10 or > 80 kg/m^2^; triglycerides <20 or > 1000 mg/dL) were excluded to improve data quality.

### 2.2. Measures

#### 2.2.1. Major Depressive Disorder (MDD)

MDD diagnosis was extracted using SNOMED concept IDs: 45557367 (MDD, single episode), 45557370 (MDD, recurrent), 440383 (depressive disorder), and 318,877 (depressive disorder NEC). Participants with any of these codes were classified as MDD-positive. We did not restrict analyses to active depressive episodes at the time of measurement, as episode timing information is not consistently available in the EHR; instead, MDD status reflects a lifetime diagnosis based on coded clinical data. Therefore, associations between mood disorder and metabolic biomarkers are interpreted in relation to MDD case status, rather than current episode severity or symptom state at the time of lab collection. Notably, we did not exclude individuals with comorbid psychiatric diagnoses such as psychotic disorders, bipolar disorder, PTSD, alcohol or substance use disorders, or eating disorders, in our primary analyses in order to preserve the representativeness and heterogeneity of the real-world clinical population sampled in the All of Us dataset. See below sensitivity analyses regarding evaluation of exclusions for psychiatric co-morbidities.

#### 2.2.2. Lipid measures (Triglycerides, HDL, and LDL)

Laboratory values for triglycerides, high-density lipoprotein (HDL), and low-density lipoprotein (LDL) were extracted based on standardized LOINC-coded tests. Triglycerides were retained if measured in serum or plasma and fell within 20–1000 mg/dL; values outside this range were recoded as missing. HDL and LDL cholesterol values were not subjected to value-based exclusions, as there is less consensus on biologically implausible ranges for these measures. As fasting status was not reliably documented in the dataset, lipid values may reflect a mix of fasting and non-fasting samples.

#### 2.2.3. Body Mass Index (BMI)

BMI values were extracted using standard concept names and filtered to retain biologically plausible values (10–80 kg/m^2^).

#### 2.2.4. Smoking frequency

Smoking behavior was assessed via self-report and coded on a 3-point scale representing current smoking frequency: 0 = “Not at all,” 1 = “Some days,” and 2 = “Every day.” This variable reflects present-day cigarette use and was used in sensitivity analyses described below to examine whether smoking status influenced observed associations among MDD, BMI, and lipid biomarkers.

#### 2.2.5. Covariates

Age was calculated using date of birth and date of measurement. Sex was included as a binary variable (“Male” or “Female”); participants with non-binary, skipped, or missing values were excluded from stratified analyses. Average alcohol intake was included as a continuous covariate. It was assessed via self-report and coded on a 5-point scale, ranging from 1 (“1–2 drinks per day”) to 5 (“10 or more drinks per day”), reflecting average daily alcohol consumption.

### 2.3. Statistical analyses

All analyses were conducted in R, version 4.3.1 (R Core Team, 2021). Descriptive statistics were computed for key variables, including body mass index (BMI), triglyceride levels, cholesterol markers, fasting glucose, and average alcohol intake, stratified by Major Depressive Disorder (MDD) diagnosis. Between-group comparisons were evaluated using Welch’s two-sample *t*-tests, which allow for unequal variances (West, 2021). In all adjusted regression models—including those used for mediation analysis—average alcohol intake was included as a covariate to account for its potential influence on both BMI and lipid levels.

In addition to *p*-values, standardized mean differences (SMDs) were calculated to quantify effect sizes for both continuous and categorical variables using the tableone package (Panos and Mavridis, 2020). For continuous variables, SMDs are equivalent to Cohen’s d and reflect differences in means scaled by pooled standard deviation. For categorical variables, generalized SMDs reflect standardized differences in proportions across levels. SMDs were interpreted according to conventional thresholds: values <0.10 indicated negligible differences, 0.10–0.20 small, 0.20–0.30 moderate, and > 0.30 large differences (Yang and Dalton, 2012). Unlike *p*-values, which are highly sensitive to sample size, the standardized mean difference (SMD) provides a stable estimate of group difference magnitude. While the precision of the SMD estimate (e.g., its confidence interval) depends on sample size, the point estimate itself is not influenced by it. As such, SMDs are commonly used in large observational studies to evaluate the magnitude and direction of group differences.

Pearson correlation coefficients were used to quantify the linear relationship between BMI and triglyceride levels. Correlation strength was interpreted based on guidelines from Schober et al. (Schober et al., 2018), commonly used in clinical and biomedical research, where values of 0.10–0.39 are considered weak, 0.40–0.69 moderate, and ≥ 0.70 strong. These thresholds differ slightly from Cohen’s (Cohen, 1988) conventions used in behavioral sciences, where correlations of 0.10, 0.30, and 0.50 are typically considered small, medium, and large, respectively. All p-values were two-tailed, with *p* < 0.05 considered statistically significant unless otherwise specified (Cohen, 1988).

Missing data patterns were evaluated using Little’s MCAR test (Li, 2013) on a random subsample of 3000 participants. The test was highly significant (*p* = 6.77 × 10^−72^), indicating that the data were not missing completely at random (MCAR). This result supports the use of multiple imputation under the more flexible missing at random (MAR) assumption.

### 2.4. Mediation analysis

To test whether BMI mediates the relationship between MDD and triglycerides, we used the mediate function from the mediation R package (Tingley et al., 2014). The mediator model regressed BMI on MDD status and covariates (age, sex, and average alcohol intake). The outcome model regressed triglycerides on MDD status, BMI, and same covariates. Mediation analyses used nonparametric bootstrapping (1000 simulations) to estimate the average causal mediation effect (ACME), average direct effect (ADE), and proportion mediated. A complete case approach was applied to ensure consistency across models and avoid biases due to differential missingness across variables.

### 2.5. Sex-stratified and moderated mediation

To evaluate whether the mediation effect of BMI on the association between MDD and triglyceride levels varied by sex, we conducted a moderated mediation analysis. This approach involved incorporating interaction terms in both the mediator model (MDD × sex predicting BMI) and the outcome model (BMI × sex predicting triglycerides), allowing for differential pathway effects by sex. We estimated average causal mediation effects (ACME) separately for males and females using the mediate() function in R, specifying sex as a covariate. Alcohol intake was not included as a covariate in this analysis to preserve statistical power for detecting moderation effects, given the added complexity introduced by interaction terms and smaller subgroup sizes. To statistically assess whether the mediation effects significantly differed by sex, we performed a nonparametric bootstrap test comparing the distributions of the ACME estimates between groups. This framework allowed for a direct test of effect modification while also presenting stratified estimates to aid interpretation.

### 2.6. Exploratory age-stratified mediation analysis

To explore whether the mediation effect varied by age, we conducted age-stratified mediation analyses using conditional inference within the mediate() framework (Tingley et al., 2014). The models matched the primary analysis in structure—including adjustment for age, sex at birth, and average alcohol intake—and specified fixed age values (25, 45, and 65 years) using the covariates argument. This allowed for estimation of the ACME, ADE, and proportion mediated at different adult life stages. These analyses were intended to probe potential developmental differences in the role of BMI as a mediator linking MDD to triglycerides.

### 2.7. Sensitivity analyses

To evaluate whether the observed mediation effect was driven by participants with serious psychiatric comorbidities, we conducted a sensitivity analysis excluding individuals with diagnostic codes for schizophrenia spectrum disorders, bipolar disorder, post-traumatic stress disorder (PTSD), substance use disorders (SUD), and eating disorders using SNOMED concept IDs. The mediation analysis was then repeated using the same model structure (BMI as mediator, MDD as predictor, triglycerides as outcome), adjusting for age, sex, and average alcohol intake, and using nonparametric bootstrapping with 1000 simulations.

In addition, to assess the robustness of the mediation findings to missing data, we conducted multiple imputation-based sensitivity analyses (Baraldi and Enders, 2010). Missing values in the primary variables— body mass index (BMI), triglycerides, age, sex at birth, and average alcohol consumption—were addressed using multiple imputation by chained equations (MICE) (Buuren and van Buuren and Groothuis-Oudshoorn, 2011). Predictive mean matching was employed to generate five imputed datasets. The imputation model included all variables from the primary mediation analysis to preserve associations among predictors and outcomes.

Within each imputed dataset, we re-estimated the mediation model examining whether BMI mediated the association between major depressive disorder (MDD) and triglyceride levels, adjusting for age, sex at birth, and average alcohol intake. The mediate() function from the R mediation package (Tingley et al., 2014) was used to compute the ACME, ADE, total effect, and proportion mediated, using 1000 nonparametric bootstrap samples per imputed set.

We then applied Rubin’s rules (Rubin, 1987) to pool point estimates and standard errors across the five imputed datasets. Final summary estimates included pooled estimates, standard errors, 95 % confidence intervals (CIs), and *p*-values for the average causal mediation effect (ACME), average direct effect (ADE), total effect, and proportion mediated. Confidence intervals that did not include zero were interpreted as statistically significant at the α = 0.05 level (Hazra, 2017). These analyses allowed us to assess whether the mediation findings were robust to potential biases introduced by incomplete data.

Finally, we conducted an additional sensitivity analysis to evaluate the influence of smoking on the observed mediation pathway. Models were re-estimated with self-reported smoking frequency included as a covariate in both the mediator and outcome models. This allowed us to assess whether the indirect effect of MDD on triglycerides through BMI remained statistically significant after accounting for smoking behavior.

## 3. Results

### 3.1. Descriptive and group comparisons (Tables 1 and 2)

Individuals with MDD (*n* = 53,258) had significantly higher BMI compared to those without MDD (*n* = 148,019; M = 31.6, SD = 7.95 vs. M = 29.8, SD = 7.32; *p* < 0.001; SMD = 0.234), reflecting a small-to-moderate effect size. Triglyceride levels were also elevated among those with MDD (M = 143.5 mg/dL, SD = 96.88) compared to individuals without MDD (M = 125.9 mg/dL, SD = 83.10; p < 0.001; SMD = 0.195).

Fasting glucose levels did not significantly differ between groups (MDD: M = 99.5 mg/dL, SD = 26.22; No MDD: M = 98.1 mg/dL, SD = 23.53; *p* = 0.342; SMD = 0.062).

HDL cholesterol was slightly lower in the MDD group (M = 53.5 mg/dL, SD = 17.17) than in the non-MDD group (M = 54.7 mg/dL, SD = 17.45; p < 0.001), but the effect size was small (SMD = 0.069). Similarly, LDL cholesterol was marginally lower in individuals with MDD (M = 98.4 mg/dL, SD = 36.39) than in those without MDD (M = 99.0 mg/dL, SD = 35.48; *p* = 0.004), with a negligible effect size (SMD = 0.017).

Participants with MDD also reported higher average daily alcohol consumption (M = 1.36 drinks/day, SD = 0.77) compared to those without MDD (M = 1.28 drinks/day, SD = 0.65; *p* < 0.001), though the effect size was small (SMD = 0.116).

BMI and triglyceride levels were positively correlated (*r* = 0.22, *p* < 2.2 × 10^−16^), indicating a modest association between adiposity and elevated triglycerides.

### 3.2. Mediation analysis (Table 3)

We next tested whether BMI mediated the association between MDD and triglycerides using nonparametric bootstrap mediation analysis (1000 simulations), adjusting for age, sex at birth, and alcohol consumption. The ACME was statistically significant (ACME = 5.46, 95 % CI [2.95, 8.17], *p* < 0.001), as was the total effect (TE = 16.00, 95 % CI [4.37, 27.58], *p* = 0.007). The ADE was not statistically significant (ADE = 10.55, 95 % CI [−0.93, 22.23], *p* = 0.084), suggesting that the effect of MDD on triglycerides may be largely mediated through BMI. The proportion mediated was estimated at 34.1 % (95 % CI [15.3 %, 117.0 %], p = 0.007), supporting partial mediation of the MDD–triglyceride relationship by BMI.

### 3.3. Stratified mediation by sex (Tables 4 and 5)

To evaluate sex differences, we conducted stratified mediation analyses. Among males (*n* = 738), the mediation effect was significant (*ACME* = 7.21, 95 % CI [0.41, 15.22], *p* = 0.036), with a non-significant direct effect and a marginally significant total effect (*p* = 0.062). In females (*n* = 1312), we observed a similar pattern: a significant *ACME* (*ACME* = 4.92, 95 % CI [2.17, 7.70], *p* < 0.001), a non-significant direct effect, and a significant total effect (*p* = 0.042).

A manual nonparametric bootstrap test comparing ACME between males and females yielded a non-significant difference (*ΔACME* = 2.54, 95 % CI [−5.33, 11.09], *p* = 1.188), suggesting no statistically significant moderation of the mediation pathway by sex.

### 3.4. Exploratory age-stratified mediation analyses (Supplementary Table 1)

To evaluate whether the strength of mediation varied across age, we conducted age-stratified mediation analyses at ages 25, 45, and 65, adjusting for sex and alcohol consumption. At age 45, BMI significantly mediated the relationship between MDD and triglyceride levels (ACME = 5.70, 95 % CI [1.49, 10.99], *p* = 0.004), accounting for approximately 32 % of the total effect (*p* = 0.008). A similar pattern was observed at age 65, where the ACME was 6.07 (95 % CI [2.84, 9.52], p < 0.001), with 33 % of the total effect mediated (*p* < 0.001). At age 25, the mediation effect was weaker and not statistically significant (ACME = 5.09, 95 % CI [−0.36, 13.35], p = 0.076). These results suggest that the mediating role of BMI in the association between MDD and triglyceride levels becomes more pronounced with increasing age.

### 3.5. Sensitivity analyses (psychiatric comorbidities, missing data, smoking)

In the restricted sample (*n* = 1550) to evaluate whether the observed mediation effect was driven by participants with serious psychiatric comorbidities, the mediation effect remained statistically significant. BMI significantly mediated the relationship between MDD and triglycerides (ACME = 5.87, 95 % CI [2.95, 9.15], p < 0.001), accounting for approximately 32.4 % of the total effect (Prop. Mediated = 0.324, 95 % CI [0.135, 0.883], *p* = 0.004). The total effect remained significant (Total Effect = 18.15, 95 % CI [6.57, 31.75], p = 0.004), while the direct effect was also significant (ADE = 12.28, 95 % CI [0.74, 26.67], *p* = 0.044). These results suggest that the mediation pathway is robust to the exclusion of individuals with severe psychiatric comorbidities.

Missing data mediation analyses using multiply imputed datasets yielded results consistent with the primary findings (Supplementary Table 2). After pooling across five imputations, the average causal mediation effect (ACME) of MDD on triglycerides via BMI was statistically significant (ACME = 3.71, 95 % CI [3.09, 4.33], *p* < 2.2 × 10^−16^). The average direct effect (ADE) also remained significant (ADE = 11.93, 95 % CI [2.90, 20.97], *p* = 9.6 × 10^−3^), with a total effect estimate of 15.65 (95 % CI [6.67, 24.63], *p* = 6.4 × 10^−4^). The proportion of the total effect mediated by BMI was approximately 24.9 % (95 % CI [12.9 %, 36.9 %], *p* = 5.0 × 10^−5^). These results suggest that the observed mediation pathway is robust to missing data and likely reflects a meaningful mechanism linking depression to metabolic dysregulation.

In a sensitivity analysis adjusting for smoking frequency (*n* = 598), the mediation effect of BMI on the association between MDD and triglycerides was no longer statistically significant. The average causal mediation effect (ACME) was 2.28 (95 % CI [−2.03, 6.89], *p* = 0.274), and the total effect was not significant (Total Effect = 13.37, 95 % CI [−6.57, 33.87], *p* = 0.200). The direct effect (ADE = 11.09, 95 % CI [−8.35, 30.16], *p* = 0.300) and proportion mediated (17.0 %, 95 % CI [−177.0 %, 195.5 %], *p* = 0.382) also did not reach significance. These findings suggest that the observed mediation pathway may be sensitive to the inclusion of smoking behavior as a covariate, though the limited sample size due to missing smoking data likely reduced statistical power.

## 4. Discussion

The current study provides evidence that BMI significantly mediates the relationship between major depressive disorder (MDD) and triglyceride levels in a large, diverse sample. This finding adds to a growing body of literature indicating that metabolic dysregulation may serve as an intermediary pathway linking mental health conditions with physical health outcomes, particularly those related to cardiovascular risk. The mediation by BMI remained robust even after adjusting for age, sex, and alcohol use, suggesting that body mass may play a central role in explaining elevated triglyceride levels among individuals with MDD.

These findings align with prior literature documenting metabolic dysregulation in individuals with depression, including elevated triglycerides, insulin resistance, and obesity (Pan et al., 2012). The observed mediation by BMI is consistent with previous work indicating that excess adiposity is a key intermediary linking depression to cardiovascular and metabolic risk (Luppino et al., 2010; Milaneschi et al., 2019). Mechanistically, chronic low-grade inflammation, hypothalamic-pituitary-adrenal (HPA) axis dysregulation, and altered health behaviors (e.g., poor diet, physical inactivity, and alcohol use) have all been implicated in the bidirectional relationship between mood and metabolic health (Dowlati et al., 2010; Vogelzangs et al., 2008).

Although some prior studies have reported sex-specific pathways linking depression to metabolic outcomes (Kuehner, 2017), our findings suggest that BMI similarly mediates the relationship between MDD and triglycerides in both males and females, with no significant differences in the magnitude of the indirect effect (ACME) across sexes. This lack of sex moderation may indicate that, despite known differences in fat distribution and hormonal regulation—where men tend to accumulate more visceral fat and women more subcutaneous fat (Karastergiou et al., 2012)—the core biological mechanisms linking adiposity to elevated triglyceride levels may operate similarly in both sexes. These mechanisms likely include leptin resistance, adipose tissue inflammation, and hepatic overproduction of VLDL, which have been implicated in both depression and hypertriglyceridemia (Grundy, 2004; Klop et al., 2013; Milaneschi et al., 2019).

In addition, depression-related behavioral changes—such as physical inactivity and emotional eating—are commonly observed in both men and women and may lead to similar increases in BMI and associated lipid disturbances (Konttinen et al., 2019; Schuch et al., 2018). It is also possible that sex differences in fat distribution may influence other lipid parameters more than triglycerides, which are more tightly linked to total adiposity and hepatic lipid metabolism (Grundy, 2004; Klop et al., 2013). Future studies incorporating imaging-based measures of fat distribution and sex hormone profiles may help clarify whether more nuanced sex-specific metabolic pathways emerge beyond those detectable via BMI and triglyceride levels.

In contrast to our hypotheses, the observed differences in HDL and LDL cholesterol between individuals with and without MDD were statistically significant but small in magnitude. These findings provide limited support for an association between depression and broader dyslipidemia beyond triglycerides. While some prior studies have reported reduced HDL and elevated LDL levels among individuals with MDD, the literature has been mixed, with several large-scale and population-based studies showing weak or non-significant associations after adjusting for confounders such as BMI, smoking, diet, and physical activity (Bharti et al., 2021; Hu et al., 2023; Lehto et al., 2008; van Reedt Dortland et al., 2013). Our results are consistent with these more conservative findings, suggesting that any depression-related alterations in HDL and LDL may be modest or context-dependent. The lack of fasting status in the All of Us dataset may also have introduced measurement variability that attenuated potential associations. Future research using standardized fasting lipid panels, longitudinal designs, and more precise measures of body composition could help determine whether HDL and LDL disturbances represent reliable metabolic features of depression.

The age-stratified mediation findings add nuance to our understanding of how depression may influence metabolic health across the adult lifespan. Prior research has suggested that the cumulative burden of depression over time—through mechanisms such as chronic stress, HPA axis dysregulation, and behavioral changes—may amplify metabolic dysregulation with advancing age (Milaneschi et al., 2019; Penninx et al., 2013). Our results are consistent with this view, showing that BMI more strongly mediated the relationship between MDD and triglyceride levels in midlife and older adults (ages 45 and 65) than in younger adults (age 25).

This pattern echoes prior studies reporting that depression-related weight gain and metabolic risk markers tend to emerge or become more pronounced in midlife, particularly among those with a chronic or recurrent course (Luppino et al., 2010). The absence of significant mediation at younger ages may reflect limited cumulative exposure or more resilient metabolic profiles earlier in adulthood. These findings emphasize the importance of developmental timing in understanding the interplay between mood and metabolism and suggest that interventions aiming to prevent cardiometabolic complications of depression may be especially impactful when initiated earlier in the life course.

Given the high prevalence of both depression and metabolic syndrome in the general population(Lemche et al., 2017), our findings underscore the importance of integrated public health strategies. Interventions that concurrently target mental health and metabolic parameters—such as behavioral weight loss programs tailored for individuals with depressive symptoms—may offer dual benefits. The bidirectional relationship between depression and obesity suggests that treating one may have spillover benefits for the other.

From a clinical perspective, these results support the routine inclusion of metabolic screening—including assessments of BMI and lipid profiles—in the evaluation and ongoing care of patients with MDD. Identifying individuals with co-occurring mental and metabolic dysregulation can facilitate targeted interventions that address both domains, potentially improving both psychiatric and cardiometabolic outcomes. Integrated care models that bring together behavioral health and primary care or endocrinology may be especially beneficial (Staab et al., 2022).

At the biological level, the mechanisms underlying this mediation pathway are likely multifactorial. Beyond excess adiposity, shared biological processes such as hypothalamic–pituitary–adrenal (HPA) axis dysregulation, chronic low-grade inflammation, altered lipid signaling, and mitochondrial dysfunction may contribute to both MDD and hypertriglyceridemia. While BMI is a useful proxy, it does not differentiate between lean and fat mass, nor does it capture visceral adiposity, which has been shown to correlate with the severity of depression (Liu et al., 2024). Future studies using more precise measures—such as waist circumference, waist-to-hip ratio, or imaging-based assessments—could refine our understanding of which aspects of adiposity are most relevant.

This study has several limitations. First, its cross-sectional design precludes inferences about causality or temporal ordering. Longitudinal mediation analyses are needed to clarify the directionality of these associations. Second, BMI was used as a general proxy for adiposity, and more refined or mechanistic measures (e.g., visceral fat, waist-to-hip ratio) may yield different or more nuanced results. Third, although we adjusted for age, sex, and alcohol consumption, other important behavioral covariates—such as physical activity, dietary intake, and psychotropic medication use—were not included due to limited availability or excessive missingness in the current dataset. Fourth, while we conducted a sensitivity analysis including smoking frequency, the sample size for this analysis was substantially reduced, leading to loss of statistical significance and suggesting potential power limitations rather than definitive attenuation of the effect. Fifth, although the sample was racially and ethnically diverse, race and ethnicity were not explicitly included in the primary mediation model. Given that both MDD prevalence and metabolic risk factors may vary by race and ethnicity due to genetic, socio-economic, and cultural factors, future models should evaluate these variables as potential moderators or confounders. Finally, although several lifestyle and behavioral variables have known influence on both BMI and lipid metabolism, the constraints of available data precluded their inclusion in the primary mediation model, limiting the internal validity of the observed effects.

In conclusion, this study provides novel evidence that BMI partially mediates the association between MDD and elevated triglycerides. These findings highlight the need to address physical health in the treatment of depression and suggest that improving weight-related outcomes could be a viable strategy for mitigating cardiometabolic risk in individuals with mood disorders. Future studies should investigate more precise metabolic and adiposity markers, assess causality through longitudinal designs, and evaluate whether targeted interventions can simultaneously improve both mental and metabolic health outcomes, while accounting for additional behavioral factors such as smoking and diet when sufficient data are available.

## Supplementary Material

1

## Figures and Tables

**Table 1:** Demographic characteristics of participants by MDD diagnosis.

Variable	No MDD (*n* = 148,019)	MDD (*n* = 53,258)	p-value	SMD
Age, mean (SD)	61.02 (15.68)	60.91 (14.67)	0.173	0.007
Sex at Birth, n (%)			<0.001	0.204
- Female	85,778 (58.0 %)	35,859 (67.3 %)		
- Male	60,887 (41.1 %)	16,725 (31.4 %)		
- Other/Unknown	1354 (0.9 %)	674 (1.3 %)		
Race, n (%)			<0.001	0.178
- White	90,040 (60.8 %)	31,475 (59.1 %)		
- Black/African American	22,614 (15.3 %)	9297 (17.5 %)		
- Asian	5244 (3.5 %)	603 (1.1 %)		
- Multiple/Other/Unknown	Remaining categories	Remaining categories		
Ethnicity, n (%)			<0.001	0.034
- Hispanic/Latino	21,504 (14.5 %)	8060 (15.1 %)		
- Not Hispanic/Latino	122,527 (82.8 %)	43,547 (81.8 %)		
- Other/Unknown	Remaining categories	Remaining categories		

Demographic characteristics of participants in the All of Us cohort stratified by Major Depressive Disorder (MDD) diagnosis. Statistically significant group differences were observed for sex, race, and ethnicity (all p < 0.001). Standardized mean differences (SMDs) indicate small-to-moderate imbalances for sex (SMD = 0.204) and race (SMD = 0.178), with minimal differences in age and ethnicity. Per All of Us Research Program policy, demographic subgroups with cell counts of 20 or fewer are suppressed to protect participant privacy; these are denoted as “Remaining categories.”

**Table 2:** Key metabolic, anthropometric, and behavioral measures by MDD diagnosis.

Variable	No MDD (n = 148,019)	MDD (n = 53,258)	p-value	SMD
BMI, mean (SD)	29.80 (7.32)	31.59 (7.95)	<0.001	0.234
Triglycerides, mean (SD)	125.87 (83.10)	143.45 (96.88)	<0.001	0.195
Fasting Glucose, mean (SD)	98.14 (23.53)	99.69 (26.22)	0.342	0.062
HDL Cholesterol, mean (SD)	54.74 (17.45)	53.54 (17.17)	<0.001	0.069
LDL Cholesterol, mean (SD)	99.00 (35.48)	98.40 (36.39)	0.004	0.017
Average Drinks per Day, mean (SD)	1.28 (0.65)	1.36 (0.77)	<0.001	0.116

Comparison of metabolic and behavioral measures between participants with and without Major Depressive Disorder (MDD). Individuals with MDD exhibited significantly higher BMI and triglyceride levels, with small-to-moderate standardized mean differences (SMD = 0.234 and 0.195, respectively). While statistically significant, differences in HDL, LDL, and average daily alcohol consumption (SMD = 0.069, 0.017, and 0.116, respectively) reflect small effect sizes. Fasting glucose differences were not statistically significant. These findings suggest modest but notable metabolic and behavioral differences associated with MDD status.

**Table 3:** Covariate-adjusted mediation of the association between MDD and triglycerides by BMI.

Effect	Estimate	95 % CI (Lower)	95 % CI (Upper)	p-value
ACME	5.455	2.950	8.170	<0.001 ***
ADE	10.547	−0.926	22.230	0.084
Total Effect	16.002	4.367	27.580	0.007 **
Proportion Mediated	0.341	0.153	1.170	0.007 **

Results from causal mediation analysis testing whether BMI mediates the relationship between major depressive disorder (MDD) diagnosis and triglyceride levels, adjusting for age, sex, and alcohol consumption (average drinks per day). Estimates are based on nonparametric bootstrapping with 1000 simulations. The average causal mediation effect (ACME) was significant, suggesting that BMI partially mediates the MDD–triglyceride relationship. The proportion mediated was 34.1 %, indicating that approximately one-third of the total effect operates through BMI.

**Table 4:** Moderated mediation analysis of BMI as mediator between MDD and triglycerides, stratified by sex.

Effect	Sex	Estimate	95 % CI (Lower)	95 % CI (Upper)	p-value
ACME	Male	7.205	0.500	14.860	0.030 *
ADE	Male	10.800	−1.161	22.530	0.076
Total Effect	Male	18.004	4.989	32.310	0.006 **
Prop. Mediated	Male	0.400	0.043	1.130	0.032 *
ACME	Female	4.836	2.335	7.790	<0.001 ***
ADE	Female	10.800	−1.161	22.530	0.076
Total Effect	Female	15.636	3.816	27.910	0.014 *
Prop. Mediated	Female	0.309	0.131	1.120	0.014 *

Stratified mediation analysis showing BMI as a mediator between MDD and triglycerides in males and females. Models adjusted for age. ACME: Average Causal Mediation Effect. ADE: Average Direct Effect.

**Table 5:** Bootstrap test of moderated mediation: sex differences in the ACME.

Comparison	Observed Difference	95 % CI (Lower)	95 % CI (Upper)	p-value
Male – Female ACME	2.540	−5.329	11.091	1.188

Manual nonparametric bootstrap test comparing the ACME between males and females to assess moderation by sex. Despite differences in point estimates, the confidence interval included zero and the difference was not statistically significant.

## Data Availability

This study used data from the All of Us Research Program Registered Tier Dataset (version 8). Access to the All of Us data is available to registered researchers via the Researcher Workbench (https://www.researchallofus.org). The data are not publicly available due to privacy and security considerations, but qualified researchers may obtain access after completing required training and agreeing to the All of Us Data and Statistics Dissemination Policy.

## Appendix A. Supplementary data

Supplementary data to this article can be found online at https://doi.org/10.1016/j.jad.2025.119889.
