# Supplementary material for: Body mass index mediates the relationship between depression and triglyceride levels: Evidence from a large national cohort
Source: J Affect Disord. Author manuscript; Available in PMC 2026 Jan 20. (PMC12817363; doi:10.1016/j.jad.2025.119889)
Supplement: 1 [file NIHMS2135223-supplement-1.docx]

**Supplementary Table 1. Age-Stratified Mediation of the Relationship Between MDD and Triglycerides via BMI**

| **Age** | **ACME (95% CI)** | **p-value** | **ADE (95% CI)** | **p-value** | **Total Effect (95% CI)** | **p-value** | **Proportion Mediated (95% CI)** | **p-value** |
| --- | --- | --- | --- | --- | --- | --- | --- | --- |
| 25 | 5.09 [–0.36, 13.35] | 0.076 | 12.18 [0.70, 26.80] | 0.044 | 17.27 [4.42, 35.81] | 0.014 | 0.29 [–0.04, 0.79] | 0.082 |
| 45 | 5.70 [1.49, 10.99] | 0.004 | 12.18 [–0.20, 25.97] | 0.058 | 17.88 [4.81, 32.31] | 0.004 | 0.32 [0.09, 1.02] | 0.008 |
| 65 | 6.07 [2.84, 9.52] | <0.001 | 12.18 [0.12, 24.60] | 0.050 | 18.24 [6.29, 31.10] | <0.001 | 0.33 [0.14, 0.98] | <0.001 |

Results of age-stratified mediation analyses estimating the average causal mediation effect (ACME), average direct effect (ADE), total effect, and proportion of the total effect mediated by body mass index (BMI) in the association between Major Depressive Disorder (MDD) and triglyceride levels. Mediation models were adjusted for sex and alcohol consumption and run separately for age values of 25, 45, and 65. Estimates were generated using 1,000 nonparametric bootstrap simulations.

**Supplementary Table 2. Pooled Mediation Effects After Multiple Imputation**

| **Effect** | **Estimate** | **Std. Error** | **95% CI (Lower)** | **95% CI (Upper)** | **p-value** |
| --- | --- | --- | --- | --- | --- |
| ACME | 3.71 | 0.32 | 3.09 | 4.33 | < 2.2 × 10⁻¹⁶ |
| ADE | 11.93 | 4.61 | 2.90 | 20.97 | 9.65 × 10⁻³ |
| Total Effect | 15.65 | 4.58 | 6.67 | 24.63 | 6.38 × 10⁻⁴ |
| Prop. Mediated | 24.88% | 6.13% | 12.85% | 36.90% | 5.01 × 10⁻⁵ |

This table presents the pooled estimates from a causal mediation analysis examining whether body mass index (BMI) mediates the relationship between major depressive disorder (MDD) and triglyceride levels. Results are based on five multiply imputed datasets (m = 5), using Rubin’s rules to combine point estimates and standard errors. The average causal mediation effect (ACME), average direct effect (ADE), total effect, and proportion mediated are all statistically significant, with approximately 24.9% of the total effect of MDD on triglycerides accounted for by BMI. These findings confirm that the mediation results are robust to potential bias introduced by missing data.
